# *FaceLooks*: A Smart Headband for Signaling Face-to-Face Behavior

**DOI:** 10.3390/s18072066

**Published:** 2018-06-28

**Authors:** Taku Hachisu, Yadong Pan, Soichiro Matsuda, Baptiste Bourreau, Kenji Suzuki

**Affiliations:** 1 Faculty of Engineering, Information and Systems, University of Tsukuba, Tsukuba 3058573, Japan; bourreau@ai.iit.tsukuba.ac.jp (B.B.); kenji@ieee.org (K.S.); 2 Empowerment Informatics Program, University of Tsukuba, Tsukuba 3058573, Japan; panyadong@ai.iit.tsukuba.ac.jp; 3 Faculty of Human Sciences, University of Tsukuba, Tsukuba 3058573, Japan; matsuda@ai.iit.tsukuba.ac.jp

**Keywords:** behavioral sciences, children with intellectual disabilities and/or autism spectrum disorders, *FaceLooks*, facing behavior, infrared sensors, wearable computers

## Abstract

Eye-to-eye contact and facial expressions are key communicators, yet there has been little done to evaluate the basic properties of face-to-face; mutual head orientation behaviors. This may be because there is no practical device available to measure the behavior. This paper presents a novel headband-type wearable device called *FaceLooks*, used for measuring the time of the face-to-face state with identity of the partner, using an infrared emitter and receiver. It can also be used for behavioral healthcare applications, such as for children with developmental disorders who exhibit difficulties with the behavior, by providing awareness through the visual feedback from the partner’s device. Two laboratory experiments showed the device’s detection range and response time, tested with a pair of dummy heads. Another laboratory experiment was done with human participants with gaze trackers and showed the device’s substantial agreement with a human observer. We then conducted two field studies involving children with intellectual disabilities and/or autism spectrum disorders. The first study showed that the devices could be used in the school setting, observing the children did not remove the devices. The second study showed that the durations of children’s face-to-face behavior could be increased under a visual feedback condition. The device shows its potential to be used in therapy and experimental fields because of its wearability and its ability to quantify and shape face-to-face behavior.

## 1. Introduction

Face-to-face communication is a fundamental social behavior involving head orientation, in which two people face one another. This state triggers (but does not include) interactions of eyes, lips, and head orientation, for example. This behavior allows two or more people to easily make eye contact [1], localize sound, observe the shape of the lips, coordinate speaker turns, and express happiness [2]. Ono et al. indicated that a high frequency of face-to-face behavior was associated with better mental health [3]. We consider that the behaviors of rotating the head and facing peers are positive social signals, which represent a person’s intent to trigger and facilitate further interaction. However, the correlation of the face-to-face behavior with other social behaviors such as eye contact is still unclear because, until now, there has been no practical device available to detect the face-to-face behavior.

The purpose of this study was to measure and shape face-to-face behavior using wearable technology for contexts such as personalized training for social communication. In the domain of social skills training for children with intellectual disabilities and/or autism spectrum disorders (ASD), face-to-face behavior plays a key role in initiating other social communications. Work settings require important job-related social skills based on face-to-face behavior such as greetings, reporting, and interactions with other workers. We aimed to achieve our purpose using a lightweight device that quantitatively measures the time and frequency of the face-to-face behavior as well as identifying the faced partner. Additionally, the device provides visual feedback when it detects face-to-face behavior. Certainly, most people can perceive whether they are face-to-face with a partner without the visual feedback. However, this is not always easy for some individuals with intellectual disabilities and/or ASD because they often avoid or do not look at the partner’s face [4,5]. Thus, by providing real-time feedback, the device allows users to become aware of their own behavior. Then, we can help modify the social behavior as needed. In this study, the device was designed for a special-needs therapy scenario. Using this device for social skills training in therapy and experimental fields is a challenge for behavioral health applications.

**Figure 1:**
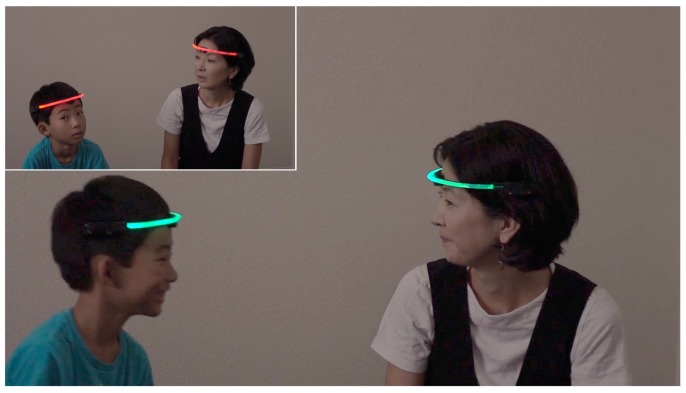
FaceLooks: wearable devices measuring and visually representing face-to-face behavior in real time.

**Figure 2:**
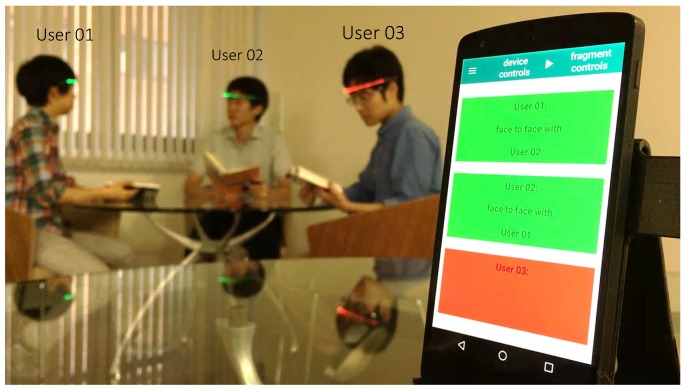
Cooperating with an Android device allows monitoring and automatic logging of face-to-face behaviors.

In this paper, we introduce the wearable device with infrared (IR) communication, called FaceLooks, which quantifies face-to-face behavior. This smart sensor device is worn on the forehead like a headband, and emits light to visually represent real-time face-to-face behavior, as shown in Figure 1. The device communicates with an Android tablet or smart phone via Bluetooth. The measured face-to-face behavior can then be monitored to assess the behavior and for automatic logging in real time, as shown in Figure 2. This allows trainers and therapists to quickly review and analyze the characteristics of the wearer’s behavior. Note that FaceLooks is free from the privacy issues caused by computer vision-based devices.

To evaluate the function and potential of the developed device, we conducted field experiments in a special needs school for children with intellectual disabilities and/or ASD in addition to laboratory experiments. We chose the special needs school for the case studies because research suggests these children participate in fewer social and recreational activities and have fewer friends than typically developed children [6]. Furthermore, the co-occurrence of intellectual disabilities and ASD is common [7], and it is often reported that children with ASD tend to avoid facing, or looking at other people’s faces [4,5].

This paper first defines physical face-to-face behavior, reviewing previous literature on extant methodologies for measuring social-spatial orientation. Next, we describe the design, principle, and implementation of the FaceLooks prototype. Three laboratory experiments validate the intended performance of the developed device. We then describe two field studies with several children from a special needs school. In the first field study, we quantitatively measured the children’s face-to-face behavior during mealtime. In the second field study, we demonstrated the effect of visual feedback for shaping their face-to-face behavior in a class where children with intellectual disabilities and/or ASD practice task completion reports. In this class, teachers instruct the children to face their peers when they make their report. Finally, we conclude the paper with our future research directions. The contributions of this research are as follows.

- Implementation of hardware and software of a headband device that quantitatively monitors and visually augments face-to-face behavior in real time, using IR communication.
- Evaluation of the performance of the device (e.g., detection range, response time, and comparison with a human coder and wearable gaze trackers) of the device in laboratory experiments.
- Conducting a field study in a special needs school to quantitatively monitor face-to-face behavior of children with intellectual disabilities and/or ASD during daily life activities, for example at mealtime.
- Conducting a field study in a class for demonstrating the effect of the visual representation for signaling the face-to-face behavior in children with intellectual disabilities.

## 2. Related Work

### 2.1. Face-to-Face Behavior and Social-Spatial Orientation

**Figure 3:**
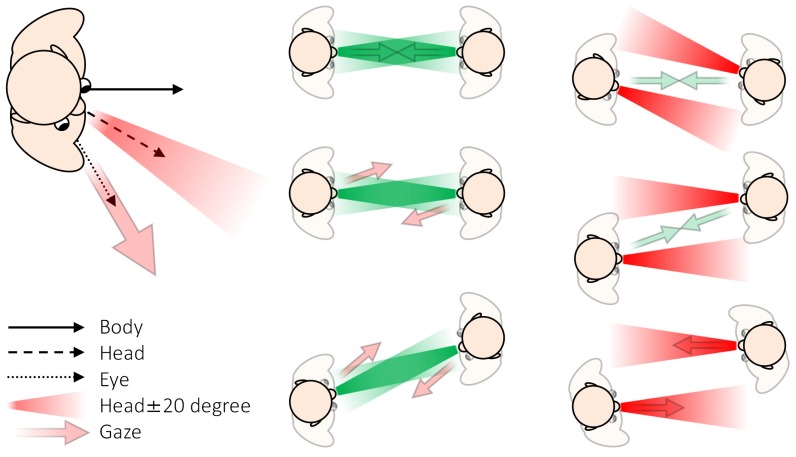
Body orientation, head orientation, eye position, and gaze position (**Left**); illustration of face-to-face behavior (**Middle**); and not face-to-face behavior (**Right**), from our definition.

In this study, we defined face-to-face behavior as a physical state in which two person’s faces are within ±20° of each other’s facing direction. Therefore, we considered only the head orientation; not body orientation, eye position, or gaze direction (sum of the eye position and the head orientation relative to the body [8,9]), as shown in Figure 3.

Interpersonal distance is a subjective measure defining a human’s personal space. According to Hall, who defined proxemics, this invisible range is one of the key dimensions of society [10]. Kendon suggested the concept of F-formation.

“An F-formation arises whenever two or more people sustain a spatial and orientational relationship in which the space between them is one to which they have equal, direct, and exclusive access [11]”.

These are sociology-oriented concepts rather than physical parameters (e.g., spatial relationships of bodies including foot layout, shoulder orientation, and head orientation).

Face-to-face behavior is different from eye contact. Eye contact is a state during which two people’s gaze positions are located around each other’s eyes or faces [5,12,13]). In our definition, eye contact is not necessarily a requirement for face-to-face behavior, even though eye contact happens in many situations of face-to-face behavior.

We defined the ±20° range based on previous work on eye–head coordination. When gaze shifts more than 30°, both eye and head movements are observed, which involves saccade and vestibulo-ocular reflexes [14,15]. Conversely, for small gaze shifts, the head movements do not contribute to the whole gaze shift, and only eye movements are observed. Fang et al. studied the relationship of the distribution of eye and head positions while watching movies on an ultrahigh-definition display [9]. The eye position was distributed mostly within a range ±20°, regardless of the direction the head faced. These studies imply that humans can pay attention to things inside ±20° around the facing direction, whereas they may choose to move the head to pay attention to the things outside of that range.

### 2.2. Measurement of Social-Spatial Orientation

To measure F-formation and analyze social interaction, Setti et al. developed a computer vision-based technique for individuating freestanding conversational groups in images [16,17,18]. Several wearable devices for measuring body (i.e., torso) position (i.e., orientation) have been developed using radio [19,20] and IR communication [21,22,23]. These technologies mainly measure torso position and orientation, which allow for analyzing social signals that shows a person’s willingness to join a group. Head orientation, on the other hand, shows the person’s willingness to have further interaction with peers because facial expression is then exposed, as we mentioned in the previous section. In this sense, we differentiate our focus of behavior from those of prior works.

To analyze a person’s attention, gaze trackers are often used to measure the gaze point. Ye et al. developed a system for detecting moments of eye contact between an adult and a child, using a single pair of gaze tracking glasses worn by the adult [13]. Eye contact was registered as an event of simultaneous, mutual face-gazing via a dyad. Speer et al. used a wearable gaze tracker and reported the gaze patterns of children, both with and without autism, while they watched a video [4]. The fixation durations of the children with ASD were less for eye regions, but more for body regions, compared to typically developing children. Wearable eye trackers are precise commercially available devices. However, they are costly and it can be difficult to persuade the children to wear them. These factors prevent us from using them in multi-party situations. Computer vision technology can offer precisely estimated gaze points from images of human faces [24,25]. However, face-to-face behavior does not always occur with eye contact, i.e., there are two types of eye contact behavior: eye contact with and without face-to-face behavior. The interaction of eyes without face-to-face behavior is easily terminated because the mass of the eyeball is smaller than that of the head. Facilitating face-to-face behavior should be important, as a first step, to increase opportunities for social interaction and to enhance engagement with peers. Although it will be beneficial to simultaneously measure torso-to-torso behavior, eye contact, and face-to-face behavior, and analyze the social function of each, integrating all these systems in one solution is beyond our scope.

Therapists for children with intellectual disabilities and/or ASD have conventionally measured face-to-face behavior for diagnosing or evaluating intervention methods by using stationary video camera(s) and then coding the video data using human observers. This method is long, burdensome, and may include observer bias. One solution is to use image-processing technology, such as Face API from Microsoft and CERT [26], but it is still difficult to measure face-to-face behavior in daily life because the cameras have blind areas and occlusion problems. Using wearable cameras is another solution. Fathi et al. presented a method for detecting social interaction in daily life activities via first-person video captured by a wearable camera [27]. However, it may still be difficult to measure face-to-face behavior of children with intellectual disabilities and/or ASD in daily life activities. One reason is resolution. Systems that precisely track head orientation require high definition images, which consumes more battery power, thus making a compact wearable system for children not viable. Another reason is privacy, where video recording of children raises ethical concerns.

To augment the cocktail party phenomenon, Watanabe et al. developed a head-mounted IR communication device, which converts sound to IR light and vice versa [28]. They provided several application scenarios in which a user could verbally interact with another user or an object by rotating the head and listening or speaking. Inspired by this work, we employed a wearable IR communication device for measuring face-to-face behavior. To identify the partner during torso-to-torso interaction, badge-type devices have been developed, which transmit IR light, encoding unique IDs (i.e., device ID) [21,22,23]. For example, Choudhury developed a shoulder-mounted wearable device with IR transmitters and a receiver [21]. While users with the devices are in a torso-to-torso position, the devices can exchange the IDs via IR communication and identify the partner. Although details of the communication are not reported, a low sampling rate (17 Hz) may be used to avoid communication collision (i.e., interference of IR light). To achieve more robust communication protocols, we implemented an automatic synchronization procedure using random intervals for transmitting and receiving (see Section 3.3.2). This procedure allows for detection of face-to-face behavior and also reports telemetry.

## 3. FaceLooks

### 3.1. Design Rationale and Concerns

We defined five design requirements for the wearable device.

First, the device should be easy to wear. This is necessary to avoid limiting the user’s movements in daily life activities. We also note that complicated mechanism for fixing the device, or heavy components, would be unacceptable and would distract the participants in an experiment.

Second, the device should measure the time and duration of face-to-face behavior with each individual partner. This is necessary to evaluate the effect of the intervention and to investigate the user’s social relationship in a quantitative manner.

Third, it should be possible to use several devices simultaneously. We are interested in social dynamics among multiple people. The devices, therefore, need to communicate with each other via an ad-hoc network because it would be too difficult to predetermine the master and slave devices.

Fourth, the device should measure face-to-face behavior in real time. This enables using feedback modalities to make the users aware of face-to-face behavior.

Finally, the device should show the feedback as a social signal around the peer’s face. We assume the pair can pay attention to each other’s faces and share the event.

We employed a headband-type wearable device that provides visual feedback on the forehead. Although the user cannot see the feedback of their own device, it is more important that the person pays attention to their peer. The visual feedback helps the person to see the peer’s face during a face-to-face interaction.

As an alternative, glasses or a goggle-type wearable device may be considered. However, the former is incompatible with ordinary glasses and the latter is generally bulky.

### 3.2. Working Principle

**Figure 4:**
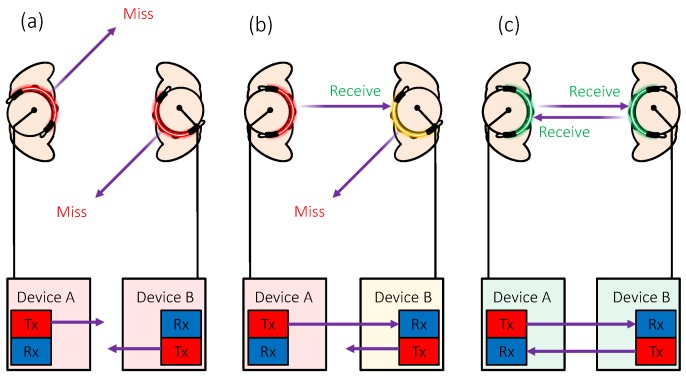
Principle of measuring face-to-face behavior by infrared communication: (**a**) the devices are not face-to-face; (**b**) Device A faces Device B while Device B does not face Device A; and (**c**) the devices are face-to-face.

We employed IR communication for measuring face-to-face behavior. The communication procedure is as follows. First, one device modulates data, including its own ID, and transmit them via an IR LED. Then, the device waits for IR light from the other device, which works in the same manner. Employing a directional IR LED, whose light axis is coaxially aligned to a receiver, the devices can establish communication (i.e., transmitting and receiving) while they are facing each other, as shown in Figure 4c. In other words, full communication cannot be established when one device faces the other while the other is facing away (Figure 4b) or when both are facing away (Figure 4a).

The devices detect face-to-face behavior when they are worn on the user’s forehead with the light axes aligned directly with the face, and they successfully communicated. Currently, the devices identify the partner by reading the ID from the received data. Furthermore, because the devices communicate with an Android device, the starting time and duration of face-to-face behavior could be monitored and logged automatically.

### 3.3. Implementation

#### 3.3.1. Hardware Components

**Figure 5:**
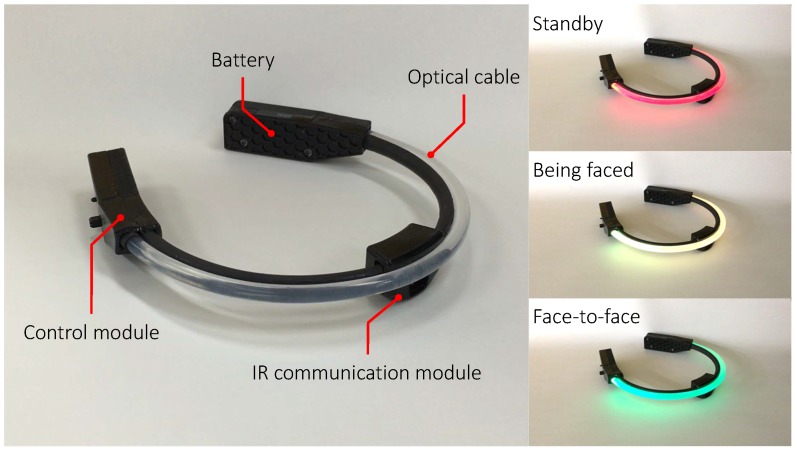
Prototype of the FaceLooks device: control module, IR communication module, optical cable, and battery fixed to a headband.

**Figure 6:**
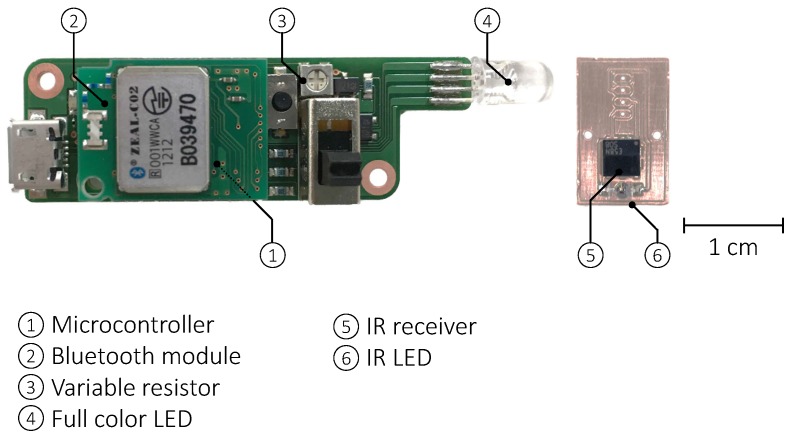
A control module consisting of a microcontroller, a Bluetooth module, a variable resistor, and a full-color LED and an IR communication module consisting of an IR receiver and an IR LED.

Figure 5 shows a prototype of the developed device, called FaceLooks. It consists of a headband, a control module, and an IR communication module. As shown in Figure 6, the control module consists of a Bluetooth module (ADC Technology, ZEAL-C02), a 1000-Ω variable resistor, a full color LED that connects to an optical cable and illuminates the headband, and a microcontroller (NXP Semiconductors, LPC11U24). The IR communication module has an IR remote control receiver module (Vishay, TSSP57038, angle of half transmission distance is ±75°), and an IR LED (Vishay, TSML1020, angle of half intensity is ±12°). The control module connects to the IR communication module and a lithium polymer battery (110 mAh). The total weight of the device is 75 g.

Using the microcontroller, the IR communication module sends a universal asynchronous receiver transmitter signal via the IR LED, which is modulated by a carrier wave using on–off keying. We employed a 38-kHz square wave as the carrier wave to fit in the bandwidth of the IR receiver. The intensity of the IR light can be adjusted by the variable resistor. The IR communication module receives IR light, demodulates the signal, and passes it to the microcontroller. We set the baud rate of IR communication to 2400, which we preliminarily confirmed as the maximum rate to ensure stable signal demodulation.

The communication range depends on the radiant intensity of the IR LED and the directivity of the IR receiver module. The former can be adjusted by the variable resistor that controls the current that flows in the IR LED, and both can be adjusted by the case of the device that limits its directivity. Through our experiments, we increased the range by around 5° from the original ±20° (see Section 4.1). Because our purpose is to signal and shape face-to-face behavior, this wider range is expected to easily draw the user’s attention.

The device cannot precisely detect face-to-face behavior while the device is out of alignment with face direction. However, fixing firmly goes against our design requirements. In the cases requiring precise measurement, we used a cap to more securely anchor the device.

#### 3.3.2. Communication Protocols

**Figure 7:**
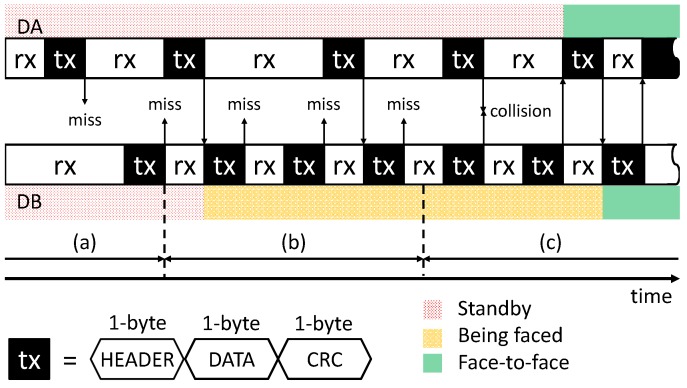
Illustration of communication procedure between Device A (DA) and Device B (DB) in Figure 4. The packet contains a header, data (e.g., device ID), and the CRC.

For bi-directional communication through single frequency IR light, we used time division to enable transmission and reception in alternating time slots (i.e., half-duplex communication). Figure 7 shows an example of the communication procedure between two devices as shown in Figure 4. A 3-byte packet (transmitted and received) consists of a 1-byte header, 1-byte data (such as device ID), and a 1-byte cyclic redundancy check (CRC).

In Phase (a), the devices are not facing each other. Each device attempts to transmit a synchronous signal with its own ID at intervals selected randomly from five alternatives (e.g., 15, 30, 45, 60, and 75 ms) to detect whether face-to-face behavior has occurred and to synchronize with other devices. Using random intervals helps avoid collisions between multiple transmitted signals. When the microcontroller is not transmitting, it waits to receive signals from other devices. In this phase, the states of both the devices are “standby” (SB).

In Phase (b), Device A faces Device B, whereas Device B does not face Device A. If the signal from Device A is received successfully by Device B, Device B responds with an acknowledgment header and its own ID. However, the packet is not received by Device A. In this phase, the state of Device A is still SB, and the state of Device B changes from an SB to a “being faced” (BF) state.

In Phase (c), Device A and Device B are facing each other. When Device A receives the packet with the acknowledgment header from Device B, Device A replies with a master header and its own ID and changes from an SB to a “face-to-face” (FtF) state. Similarly, when Device B receives the packet with the master header, it replies with a slave header and its own ID and changes from BF to FtF.

The master device, Device A, then leads the communication, allowing information to be exchanged. When the face-to-face behavior ends, the communication channel is terminated, and the devices return to their initial states. By this method, the devices not only detect face-to-face behavior, but they also identify the partner by receiving its ID. The sampling rate of the prototype device is 100 Hz.

To reduce jitter of transition between the three states, the device applies a filter with a window size of 50 samples, and the state is transmitted. The color of the LEDs changes according to the output. When the state is SB, BF, or FtF, it cycles between red, yellow, and green, respectively, as shown in Figure 5 and Video S1. In addition, the device communicates with an Android device via Bluetooth serial communication. The device also sends measured face-to-face behavior (i.e., the output and partner’s ID). In our current system, starting times and durations of the behavior are tagged by the Android device when it receives the event data.

## 4. Device Performance Evaluation

This section describes three laboratory experiments conducted to evaluate the basic performance of the FaceLooks prototype device. First, we measured the device’s detection ranges and response times with two dummy heads. Then, the devices were worn by three participants who played a card game. We compared the face-to-face behavior measured by the device to that measured by a human video coder and gaze trackers.

### 4.1. Detection Range

We measured the range in which the device could detect the three states (i.e., SB, BF, and FtF).

#### 4.1.1. Setup

**Figure 8:**
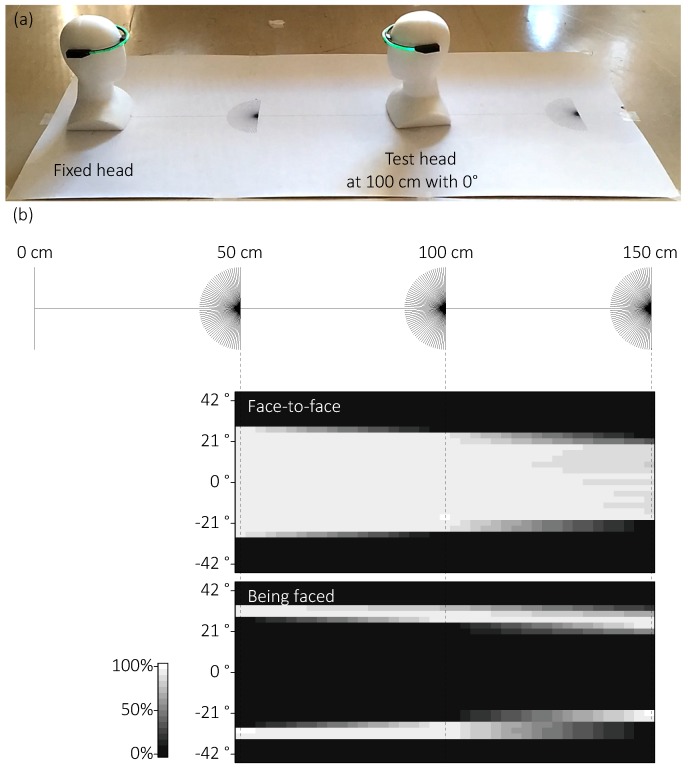
Evaluation of detection range: (**a**) A pair of dummy heads with our prototype devices were placed on a paper with a 150-cm ruler with 50-cm markings, and three protractors with 3° resolution. One head (**left**) was fixed, and the other (**right**) test head was rotated in 3° steps. We recorded the duration of the states of the device on the test head under three distances: 50, 100, and 150 cm. (**b**) Frequency of two states (BF, FtF) vs. angle of the test head for 60-s measurement (data values between the protractors are linearly interpolated). The 33.3% threshold for detecting FtF under 50, 100, and 150 cm conditions are 27.0°, 24.0°, and 18.0°, respectively.

As shown in Figure 8a, we used a pair of dummy heads with the prototype devices on white paper measuring 180 × 84 cm. We printed a 150-cm ruler with 50-cm marking and three protractors of 3° resolution at 50-cm intervals. We measured the range as follows.

1. Have the head fixed at the left end of the ruler to face the right end and have the other (test head) placed at the center of a protractors at {50, 100, or 150} cm to face the left end.
2. Start IR communication of the devices with the device on the test head sending its state (i.e., SB, BF, or FtF) to the Android device for 60 s.
3. If the Android device recorded a 60-s SB state for three trials in a row, go to the next procedure. If not, rotate the test head in a {clockwise (positive) or counter-clockwise (negative)} direction in 3° increments and go back to the Procedure 2.
4. If there is still another condition, go back to Procedure 1 with a new condition. If not, end the experiment.

There were six conditions (three protractor conditions (50, 100, and 150 cm) × two rotation directions (i.e., clockwise and counter-clockwise). We employed these distances of the protractors because daily conversation is generally conducted at a distance from 45-to-120 cm [10,29]. As mentioned, the intensity of the IR LED affects the communication range. In this evaluation, we set the current in the IR LED to 25 mA.

#### 4.1.2. Results and Discussion

Figure 8b shows the results. We defined the threshold range as the average of the absolute angles of both positive and negative directions in which the device can detect more than 33.3% of face-to-face behavior (i.e., chance rate). The threshold ranges at 50, 100, and 150 cm were 27.0°, 24.0°, and 18.0°, respectively.

Compared to our definition, the results indicate that the threshold ranges almost cover the range, that is, ±20°, under the daily conversation distances, previously stated as 45–120 cm [10,29]. The greater is the distance, the narrower is the threshold. Furthermore, the frequency of face-to-face state decreased by 3–12% at 150 cm, even when the device was within the threshold range, while the durations were more than 95% under the 50- and 100-cm conditions. One reason for these results may be the attenuation of IR light from the IR LED.

### 4.2. Response Time

We measured the response time of the device and defined it as the time interval between the initiation of physical facing and the transition of the device’s output.

#### 4.2.1. Setup

We measured two types of response times for *T_BF_* and *T_FtF_* states.

We used the same setup as in the previous evaluation. For *T_BF_* and *T_FtF_*, we put the test head at 100 cm at 27° and 0°, respectively. In this experiment, when a tact switch of the device on the test head was pressed, the device set an I/O pin from low to high while simultaneously resetting the baud rate of the IR communication to 2400. Additionally, when the device transited from SB to either BF or FtF states, the device set another I/O pin from low to high. An oscilloscope measured the time interval between the rising edges of the two I/O pins. We measured the response time as follows.

1. Quadruple the baud rate of the IR communication of the device on the test head, that is, from 2400 to 9600.
2. Press the tact switch.
3. Record the time interval displayed by the oscilloscope.
4. If the measurements for each response time (*T_BF_* and *T_FtF_*) are repeated 50 times, end the experiment. If not, go back to Procedure 1.

In Procedure 1, the device on the test head was not able to communicate with the device on the fixed head, even when they were facing each other. In Procedure 2, the devices initiated communication, as described in Figure 7. We treated the time interval displayed by the oscilloscope as the response time.

#### 4.2.2. Results and Discussion

Table 1 shows the means and the standard deviations of *T_BF_* and *T_FtF_*. Regardless of the target state, the response time was approximately 350 ms. Thus, it is not possible to measure less than 350-ms face-to-face behavior with the device. However, this latency is appropriate for our purpose, shaping face-to-face behavior because the wearers should maintain the behavior to see the visual feedback. Table 2 shows the summary of the specification of the device.

### 4.3. Comparison with Human Coder and Gaze Trackers

We compared the devices to a human video coder and wearable gaze trackers. There were three objectives of this evaluation. The first was to observe the agreement between the measurements of devices and the human coder. Note that we do not consider the data from the coder as ground truth because coding is based on the coder’s judgment, and it is unclear what kind of factors may bias the coder when judging behaviors. It is possible to observe the potential of the device, however, careful interpretation of the actual result is required. The second objective was to study the difference between face-to-face behavior and eye contact. Whereas the correlation between eye and head movement during social interaction is unknown, the setup of the experiment can affect the gaze and head behavior. Thus, the experiment was conducted in a specific scenario. The last objective was to evaluate the efficiency of the device via data analysis.

#### 4.3.1. Setup

One female and two male participants (age: 23–35 years) took part in the evaluation study. As shown in Video S1, the three participants (P1, P2, and P3) sat in front of a table 120 cm from each other. The FaceLooks devices were worn over their caps. During this experiment, we did not use the full color LED. In addition to the devices, P1 and P2 wore wearable gaze trackers (SensoMotoric Instruments, Eye Tracking Glasses 2 wireless). They were asked to play a card game called “Bicycle Old Maid”, which involves drawing a card from another player. Four stationary video cameras recorded the game and the videos were used by the human coder to determine face-to-face behavior.

The experimenter explained the rules to the participants and calibrated the devices’ positions and the gaze tracking cameras. Next, the experimenter started recording the gaze trackers and the devices using an Android device. The participants then started playing the card game. Afterwards, the experimenter stopped recording. The experiment lasted approximately 4 min.

The collected data consisted of three participants’ starting times, durations, and the partners of all face-to-face behaviors (measured by the FaceLooks devices and human video coder), and the two participants’ (i.e., P1’s and P2’s) starting times and durations of eye contact (measured by the gaze trackers). The data obtained by the FaceLooks devices were logged in the Android device. We recruited a professional human coder with seven years of experience in video coding to check whether the participants were face-to-face with their peers. The gaze tracker had three cameras. Two measured eye position, and the other recorded the first-person-view that reflects head orientation. The gaze tracker output the first-person-view video with a gaze point. The video frame in which two participants’ gaze points located within each other’s faces was coded as eye contact.

#### 4.3.2. Results and Discussion

**Figure 9:**
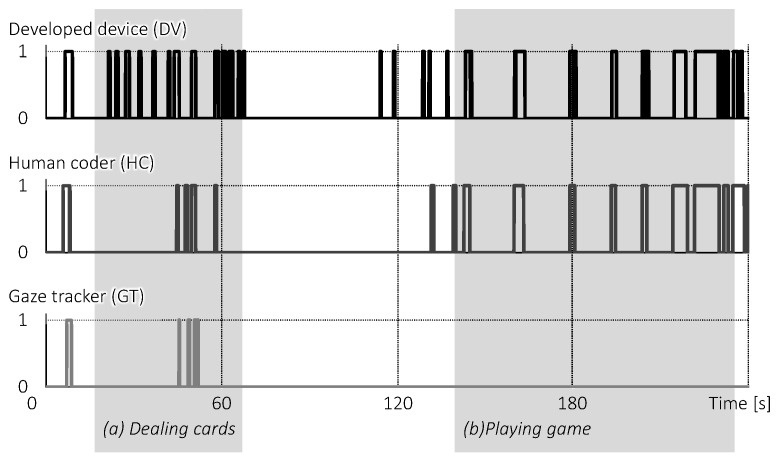
Comparison of the FaceLooks devices (FL1 and FL2) with a human video coder (HC) and wearable gaze trackers (HT): four graphs (x- and y-axes denote standby state (0)/face-to-face state (1) and time, respectively) show the data obtained from the developed device on participants A and B, the human video coder, and the pair of gaze trackers. In Periods (a) and (b), P1 dealt the cards and the others played the game.

For the following analysis, we did not consider the face-to-face events involving P3 because P3 did not wear a gaze tracker. Figure 9 shows the data obtained from the FaceLooks device on P1 and P2 (FL1 and FL2, respectively), the human coder (HC), and the pair of gaze trackers (GT). Note that the data of FL1 and FL2 were slightly different because of the protocol of the IR communication and delay induced by Bluetooth data buffering. Therefore, we computed FLAND (the logical AND of FL1’s data and FL2’s data) for the following analysis.

In addition to the total period of 240 s, we picked up two periods of the game: (a) dealing cards; and (b) playing the game. To statistically analyze the agreement of all pairs of data, we computed Cohen’s kappa coefficients for the total period, Periods (a) and (b), as shown in Table 3.

For the total period, the pair of FLAND and HC shows substantial agreement, whereas the pair of FLAND/HC and GT show slight agreement. One explanation for the results is that the two behaviors measured are independent of each other. This is because of what the FaceLooks devices and the HC measured; face-to-face behavior, is different from what the gaze tracker measured; eye contact.

During Period (a), however, the kappa coefficient of FLAND and HC was low. This may be because the distance between the faces of P1 and P2 was relatively close during card-dealing. As shown in the evaluation of the detection range, the closer the distance is, the wider the detection range becomes (Figure 8). Additionally, there was a possibility that the HC’s criterion for judging face-to-face behavior would become stricter when the distance drew closer. Therefore, the FaceLooks detected a greater amount of face-to-face behavior than the HC. One solution to increase agreement was dynamically adjusting the sensitivity of the IR receiver, or using a software filter according to the intensity of IR light. However, we consider that the device should focus on physical consistency rather than the human perceptual consistency. Thus, we did not modify the intensity of IR light.

During Period (b), the pair of FLAND and HC showed much higher agreement. However, eye contact measured by GT did not appear at all. The participant reported afterwards that he had tried to avoid gazing at the others’ faces because he was too aware of the presence of the experimenter, who was trying to monitor his gaze.

The total time taken for the HC to code the video was around 3 h. We can expect that coding time would be much longer if the experiment had lasted longer, or if the number of participants had been increased. However, the FaceLooks devices automatically coded the face-to-face behavior in real time.

In summary, the developed device can measure face-to-face behavior with a similar precision to a HC within a critical range, and it is time-efficient. Additionally, eye contact and face-to-face behavior are independent of each other. As previously mentioned, changes of experimental setup (e.g., task, participants, and HC) may affect the results. The changes strongly affect participants’ behavior and the HC’s subjectivity. We plan to investigate such an effect in our future work.

## 5. Field Study

This section describes two field studies conducted at the Special Needs School at Otsuka (Bunkyo, Tokyo, Japan). The children and their teachers participated in the experiments. Both experiments were conducted with the approval of the institutional review board of University of Tsukuba.

### 5.1. Quantitative Measurement of Face-to-Face Behavior among Children with Intellectual Disabilities and/or ASD during Activities of Daily Life

The objectives of the first field study were to observe the children’s acceptance of the device and to quantitatively measure their face-to-face behavior during activities of daily life (ADL). The experiment was conducted during mealtime. At the school, several children and teachers have meals together at a table with chairs arranged in a circle, where they randomly chat with each other, unlike during class time. Thus, mealtime is an appropriate situation in which to measure face-to-face behavior between multi-party’s ADL. Duration of the measurement, however, was limited to approximately 10 min, which is about the time taken for one of the children to finish a meal.

#### 5.1.1. Participants

As shown in Table 4, three boys and three girls (C1–C6; chronological age (CA): 13–14 years with mild/moderate intellectual disabilities and/or ASD), along with two male teachers and two female teachers (T1–T4; age: 20–50 years) participated at mealtime. The tables and chairs were arranged in a circle of approximately 250-cm diameter.

#### 5.1.2. Study Protocol

After serving the meals, caps with FaceLooks devices were worn by the participants. Once the participants started eating, the experimenter started recording the device’s output using two Android devices: one for the six devices that the children were wearing, and another for the four devices worn by the teachers. After one of the participants finished eating, the experimenter stopped recording and removed the devices from the participants. We also recorded the experiment using three stationary video cameras.

The total recording time was approximately 10 min. During the experiment, the full color LED feedback of the devices was turned off. The collected data included qualitative observation of the children’s behavior and the starting times, durations, and partners of the face-to-face behavior, as recorded by the devices.

#### 5.1.3. Results and Discussion

None of the children removed their device during the mealtime. However, some reported that the device was too small to wear for long periods. This may be because we prepared only one size of the caps fitted with FaceLooks. Additionally, the device was too big for T4 and slipped down slightly, as confirmed in the video. As can be seen from the following results, this reduced the detection of T4’s face-to-face behavior.

**Figure 10:**
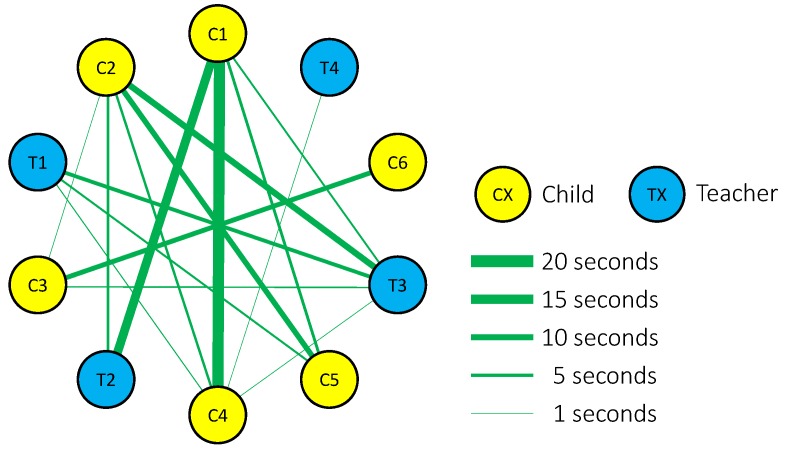
Correlation diagram made from the data obtained from the devices: the yellow and blue circles show the children and teachers, respectively; the thickness of the green line represents the duration of face-to-face behavior.

From the obtained data, we made a correlation diagram (Figure 10). The figure shows that the participants were face-to-face with the peer who sat on the facing table. However, participants at opposing ends of the table would coincidentally face each other when raising their heads, as observed by the video recording, thus it is inaccurate to characterize this event as social behavior.

When the person on the opposing end of the table was excluded, the total duration of face-to-face behavior was less than 4 s for the entire 10-min mealtime duration. Although several conversations between the children and the teachers were observed, face-to-face behavior was seldom observed, as expected.

### 5.2. Facilitating Face-to-Face Behavior

The objective of this experiment was to study whether the visual feedback provided by FaceLooks can shape face-to-face behavior in children with intellectual disabilities and/or ASD.

#### 5.2.1. Participants

The experiment was conducted during a class in which seven children and four teachers, who did not participate in the previous experiment, practiced a task completion report. In this class, interaction between a child and a teacher is mainly one-on-one basis, in which face-to-face behavior is likely to happen. Participants’ information is provided in Table 4. Two boys and two girls from the seven children (C1–C4; CA: 15–17 years with mild/moderate intellectual disabilities and/or ASD), along with three male and one female teachers (T1–T4; ages: 20–50), wore caps or bandanas with the devices at the beginning of the class. It was difficult to persuade three of the children to wear the device, probably because of oversensitivity to tactile input [30].

#### 5.2.2. Study Protocol

The experimenter recorded the output of all the eight devices using two Android devices: one for the four devices worn by the children and the other for the four devices worn by the teachers. The recording consisted of two 10-min periods. The visual feedback was not provided in the first period while the Android devices logged the events. After the first period ended, the second period began immediately. The Android devices ordered the devices to turn on the feedback function (green light) and started logging the event during the second period. When the class was finished, the experimenter removed the devices from the participants. We also recorded the experiment using three stationary video cameras.

The collected data included starting times, durations, and partners of the face-to-face behavior recorded by the devices. We did not consider the face-to-face behavior between the teachers. We did not explain the function of the devices to the children in advance to reduce bias toward the face-to-face behavior.

#### 5.2.3. Results and Discussion

**Figure 11:**
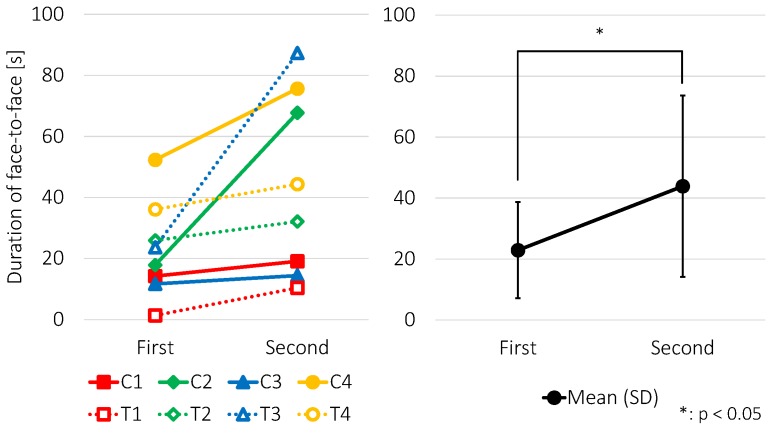
(**Left**) Total durations of face-to-face behavior in each participant for the two periods; and (**Right**) the means with standard deviations.

The total durations of face-to-face behavior of the eight participants in each of the two periods, and the means and the standard deviations, are shown in Figure 11. We performed a paired t-test on the results, and found a significant increase in the duration of the face-to-face behavior (t(7) = −2.56; *p* < 0.05).

Although durations of face-to-face behavior of all the participants increased, some of the children seemed to be unaware of the visual feedback rule. This may be because the experimenter did not explain the function of the device. However, the children who discovered the rule showed positive behavior, such as expressions of surprise and smiles. Therefore, we believe it will be more effective to explain the function of the visual feedback to shape the face-to-face behavior more successfully.

## 6. Discussion

### 6.1. Effectiveness of the FaceLooks Devices

We recorded activities of participants using stationary video cameras and confirmed that FaceLooks successfully measured the face-to-face behavior without disturbing normal activities. We noted considerable difficulties in tracking all participants’ head orientations. One reason was that checking the participant’s head orientation from all the frames by a coder required a significant amount of time, especially when the number of cameras was increased. Another reason was the limitations of the cameras (e.g., occlusion, dead angle, lighting conditions, and resolution). Therefore, the FaceLooks device can help reduce the analytical load involved in observing face-to-face interactions.

The second field study confirmed a significant increase in the face-to-face behavior under the visual feedback condition. Although some of the children were unaware of the visual feedback rule, the total duration of face-to-face behavior increased. Whereas we did not examine whether the device directly could encourage/facilitate social interaction, it is promising that the increase of these facing behaviors could provide opportunities for social interaction (e.g., eye-contact, facial expressions, smiles). Furthermore, we need to study whether this effect is induced by initial curiosity and how long the effect remains after turning off the visual feedback. We plan to consider other representation methods, such as audio and vibrational feedback, to develop more interactive protocols as means of sustaining children’s interest. Particularly, for notifying a faced state (not provided in the field studies), the audio or tactile feedback could be more useful because the wearers cannot see the visual feedback of their own device.

Our major finding from the experiment is that our device can also be effective for training purposes. Although the field study was conducted during a regular class, we consider the design of the experiment to be more research-oriented than therapy-oriented because no instruction about the visual feedback was provided to reduce bias toward face-to-face behavior. One of the teachers reported that it would be more effective if we instructed the children on the visual feedback rule. For example, by telling children “if you look at a partner’s face, the device illuminates,” and interrupting after every illumination, the children may be more likely to learn the desired behavior from contingency.

### 6.2. Limitations and Possible Solutions

During the two field studies, FaceLooks devices were worn by 18 participants, both children and teachers in the special needs school. It is possible that they could have continued wearing the devices. However, it is common that children with ASD may have oversensitivity to wearable artifacts [30]. Some of the children reported that the device was too small to wear for long periods. However, one teacher commented that the students whom the teachers expected to have difficulty wore it well, and showed interest in the device. Although there is still room for improving wearability for longer use, the devices were generally accepted during the experiments, for a period of less than 1 h. Additionally, the teacher commented that the intensity of the visual feedback was weak, and that if the visual feedback was brighter, the children would pay more attention. However, we consider that brighter visual feedback could be too intrusive and might disrupt other interactions. Whereas we did not observe such disruption with the current device, there may be a tradeoff between ambient feedback and attention.

The device cannot measure face-to-face behavior with a person without another device. Thus, all the participants must wear the devices.

The device cannot detect face-to-face behavior with more than one person. Although it was rarely found in this work, it is possible for a wearer to be face-to-face with more than one person simultaneously. Thus, the output of the device exhibited chattering among the three states with different partners’ IDs, with a quick flicker of visual feedback, using the current software. This can also happen if three devices are in close range, synchronizing with each other. In this situation, all the devices first request and wait for synchronization at random intervals. Then, two of the three devices succeed with synchronization, but the other device’s IR light breaks the synchronization instantly. Thus, all the devices return to the initial state with a pair of devices randomly succeeding in synchronization again. This process could repeat a few times within one second and appear as chattering. However, detecting this chattering state typically means detecting more than two people’s simultaneous face-to-face behavior. To suppress flicker, one solution is applying a low-pass filter to the visual feedback.

It is not possible for a wearer to see the visual feedback from his/her own device. This is because we designed the device not to present the visual feedback to its wearer to let the user avoid seeing him/herself, but let the user pay attention to the partner’s face. One solution is to employ audio and vibrational feedback as mentioned before.

It is possible for a wearer to misunderstand that they are face-to-face with a person who is actually face-to-face with someone else. For example, User A sees the green light on User B and thinks that User A is face-to-face with User B. However, User B is face-to-face with User C. While this did not happen often in this study, it would not necessarily be a limitation of the device. We consider that it may be effective for User A, and especially for children with intellectual disabilities and/or ASD, to learn about their peers facing other people.

The agreement between the device and HC is poor when the distance is relatively close. One solution is to dynamically change the sensitivity of the IR receiver or software filter, depending on the intensity of the received IR signal. In this study did not consider this in order to prioritize physical consistency rather than human perceptual consistency.

Measurement precision deteriorates when the device is not aligned with the face direction. Therefore, calibrating, positioning, and fixation of the device are important. Currently, we manually calibrate the position using visual feedback from the device or the Android device, which is one of the factors contributing to reduced precision. We can prepare various sizes and consider more flexible methods of fixation. The current device is fixed on the head by a spring force on three points. Increasing contact area distributes the pressure, making the device more comfortable.

The devices falsely detected face-to-face behavior when the wearers were standing close to walls. This was because the IR light received by the peer device was reflected from the wall. One solution would be setting the intensity of the IR LED to low, but the communication distance would then be shortened.

## 7. Conclusions

In this paper, we introduced FaceLooks, a novel headband-type wearable device for signaling the face-to-face behavior between people for behavioral healthcare applications. The device quantitatively measures face-to-face behavior during daily life activities, provides real-time visual feedback to shape the interaction, and records the starting times, durations, and identities of the facing partners in cooperation with an Android device. We quantitatively evaluated the detection range and response time of the device using three laboratory experiments, and showed substantial agreement with a human video coder for measuring face-to-face behavior. We also conducted two field experiments involving children with intellectual disabilities and/or ASD. The experiments quantitatively showed the significant increase of the durations of the children’s face-to-face behavior under the visual feedback condition.

We foresee three directions for future research. The first direction is to improve the design of FaceLooks. There is room for improving wearability for longer use. Additionally, we will also implement audio and vibrational feedback to solve the limitation of the users not being able to see the visual feedback from their own device. We will conduct a usability test to validate the device and the proposed modifications.

The second direction is to establish an intervention for mainly training the face-to-face behavior by using FaceLooks. We plan to show the devices and the results obtained by this study, to teachers and therapists, and to discuss better intervention scenarios with personalized information regarding face-to-face behavior.

The third direction involves a behavioral healthcare application for shaping and coaching a behavior chain regarding social interaction. We believe that facilitating face-to-face behavior offers the opportunity for initiating other interactions, for example greetings and handshakes. These interactions consist of a sequence of behavior. In such therapeutic activities, we can also use other wearable devices for several coaching strategies to facilitate person-to-person interaction in addition to just using the proposed device. It would be possible to support face-to-face handshakes, smooth face-to-face conversation, and face-to-face smiling by combining FaceLooks with the wearable devices that support physical touch interaction [31], smooth conversation [32], and smiling [33], respectively.

We believe that the developed device will lead to new research associated with prior studies on eye contact, smile, and conversation, recognized as a large field of investigation of person-to-person interaction.

## Figures and Tables

**Table 1:** Evaluation of response time: the standard deviations (SDs) of the device’s response times to physically being faced state (*T_BF_*) and face-to-face state (*T_FtF_*) of the test head.

	*T_BF_*	*T_FtF_*
Mean (SD) (ms)	351.75 (144.77)	310.27 (97.54)

**Table 2:** Specification of the developed FaceLooks device.

**Size**	Diameter: 15 cm
	Height: 2 cm
**Weight**	75 g
**Battery**	Feedback on: 1–2 h.
	Feedback off: 3–4 h
**Detection range**	FtF: ±18.0–27.0°
	BF: FtF + 10°
**Response time**	350 ms

**Table 3:** Comparison with human coder and gaze trackers: Cohen’s kappa coefficients for the total period, Periods (a) and (b).

		HC	GT
	Total	0.634	0.067
**FLAND**	Period (a)	0.233	0.013
	Period (b)	0.817	0.001
	Total	-	0.050
**HC**	Period (a)	-	0.126
	Period (b)	-	0.097

**Table 4:** Participants in the field study.

		*n*	Chronological Age (Years)
**Experiment 1**	Children (C1–C6)	Male = 3	13–14
		Female = 3	
	Teachers (T1–T4)	Male = 2	20–50
		Female = 2	
**Experiment 2**	Children (C1–C4)	Male = 2	15–17
		Female = 2	
	Teachers (T1–T4)	Male = 3	20–50
		Female = 1	

## Supplementary Materials

The following are available online at http://www.mdpi.com/1424-8220/18/7/2066/s1, A video abstract of this work (MP4 format movie clip, labeled Video S1) is attached.
